# Foam-based Left Atrial Appendage Closure (CLAAS) Device: Evaluation in a Chronic Canine Model

**DOI:** 10.1016/j.jscai.2022.100555

**Published:** 2022-12-01

**Authors:** Aaron V. Kaplan, Carol Devellian, Jamie H. Kim, Jessica Rotschafer, Andy Levine, Robert J. Sommer, William A. Gray

**Affiliations:** a Heart & Vascular Center, Dartmouth Hitchcock Medical Center, Lebanon, New Hampshire; b Conformal Medical, Inc, Nashua, New Hampshire; c Catholic Medical Center, Manchester, New Hampshire; d American Preclinical Services, LLC, Minneapolis, Minnesota; e Columbia University Medical Center, New York, New York; f Lankenau Heart Institute, Wynnewood, Pennsylvania

**Keywords:** atrial fibrillation, CLAAS, left atrial appendage closure, left atrial appendage occlusion

## Abstract

**Background:**

The Conformal Left Atrial Appendage System (CLAAS) is a transcatheter implant designed to occlude the left atrial appendage to prevent stroke in patients with nonvalvular atrial fibrillation. The implant incorporates a novel polyurethane polycarbonate-urea foam matrix to enhance device performance. The objective of this study was to characterize the thrombogenicity, sealing, implant integrity, local tissue response, and systemic toxicity of the implant in a healthy canine model.

**Methods:**

Devices were evaluated in a chronic canine model using echocardiographic and fluoroscopic guidance (n = 8). Postprocedural echocardiographic evaluation was performed at case completion and at 2, 45, 90, and 150 days. Animals were sacrificed at 90 (n = 4) and 150 (n = 4) days. Necropsy included gross, x-ray, and histologic examination.

**Results:**

CLAAS implants were implanted in all animals successfully on the basis of fluoroscopic, intracardiac echo, and transthoracic echo criteria. Although 2 animals were noted to have leaks (1 at implantation and 1 at day 90 post procedure before sacrifice), both were of <5 mm. All left atrial appendages were completely sealed on gross examination. No device-related thrombus, pericardial effusion, or evidence of distal organ emboli was observed. All implants maintained their structural integrity and were well integrated within the tissue. There was no histologic evidence of toxicity noted in the downstream organs or tissues.

**Conclusions:**

The CLAAS implant can be deployed providing a good seal for up to 150 days without signs of local or systemic toxicity in a canine model.

## Highlights

• Left atrial appendage closure is an emerging strategy for stroke reduction.
• Conformal Left Atrial Closure System is a next-generation foam-based device.
• Chronic canine studies showed complete closure at 90 and 150 days.

## Central Illustration

**Figure:**
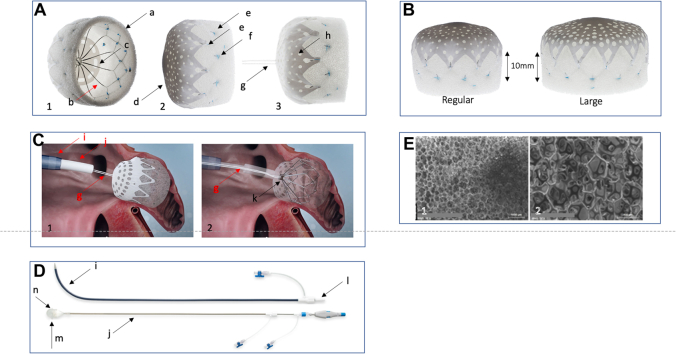

## Introduction

Left atrial appendage occlusion (LAAO) is an established stroke prevention strategy for patients with nonvalvular atrial fibrillation.^1^, ^2^, ^3^, ^4^, ^5^, ^6^, ^7^, ^8^, ^9^, ^10^ Although there are 2 commercially available devices in the United States, there are numerous devices in development that are focused on improving the implantation procedure and quality of left atrial appendage (LAA) seal.^11^, ^12^, ^13^, ^14^, ^15^ The Conformal Left Atrial Appendage System (CLAAS) (Conformal Medical) is designed to provide a soft implant with 2 sizes required to address the large range of appendage anatomy and to simplify the LAAO procedure.^14^^,^^16^^,^^17^ The conformability and sealing properties of the CLAAS implant are facilitated by the use of a novel porous foam matrix, chosen to facilitate sealing and promote tissue ingrowth. The foam provides a tissue scaffold, a compressible sealing layer along the sides of the implant, and a soft, atraumatic distal bumper to protect the LAA during implantation. The objective of this study was to assess the performance and biologic response of the implant in a canine model. More specifically, the study was designed to evaluate the thrombogenicity, sealing, implant integrity, local tissue response, and systemic toxicity of the CLAAS device in a nondiseased canine model of LAA occlusion at 90 and 150 days following implantation.

## Materials and methods

### CLAAS LAA implant and materials

The CLAAS implant is designed to eliminate blood flow into, and clot passage from, the LAA using a nitinol endoskeleton embedded in a polyurethane polycarbonate-urea foam matrix cup covered by an expanded polytetrafluoroethylene (ePTFE) cover. The highly porous open-cell structure of the foam matrix has pores of 400- to 600-μm size and has been previously studied in the vascular system.^18^ The implant is capable of being loaded into a delivery catheter and recaptured into a sheath during the implantation procedure using a tether attachment. A fenestrated ePTFE cover is attached over the proximal face to protect the foam during loading and recapture and to present a smooth, thromboresistant surface to the blood in the left atrium (LA). Fenestrations in the ePTFE cover facilitate blood flow through the implant when exposed to arterial pressures in the event of an embolization to the arterial system, for example, the distal abdominal aorta. The nitinol endoskeleton provides radial strength for implant self-expansion, anchors that secure it within the LAA and a pin at the proximal end to secure the implant to the delivery system via a flexible tether. Radiopaque markers, fabricated from platinum-iridium (90% platinum and 10% iridium), delineate the distal end of the implant and are used to help facilitate the deployment using fluoroscopic imaging (Central Illustration).

**Central Illustration:**
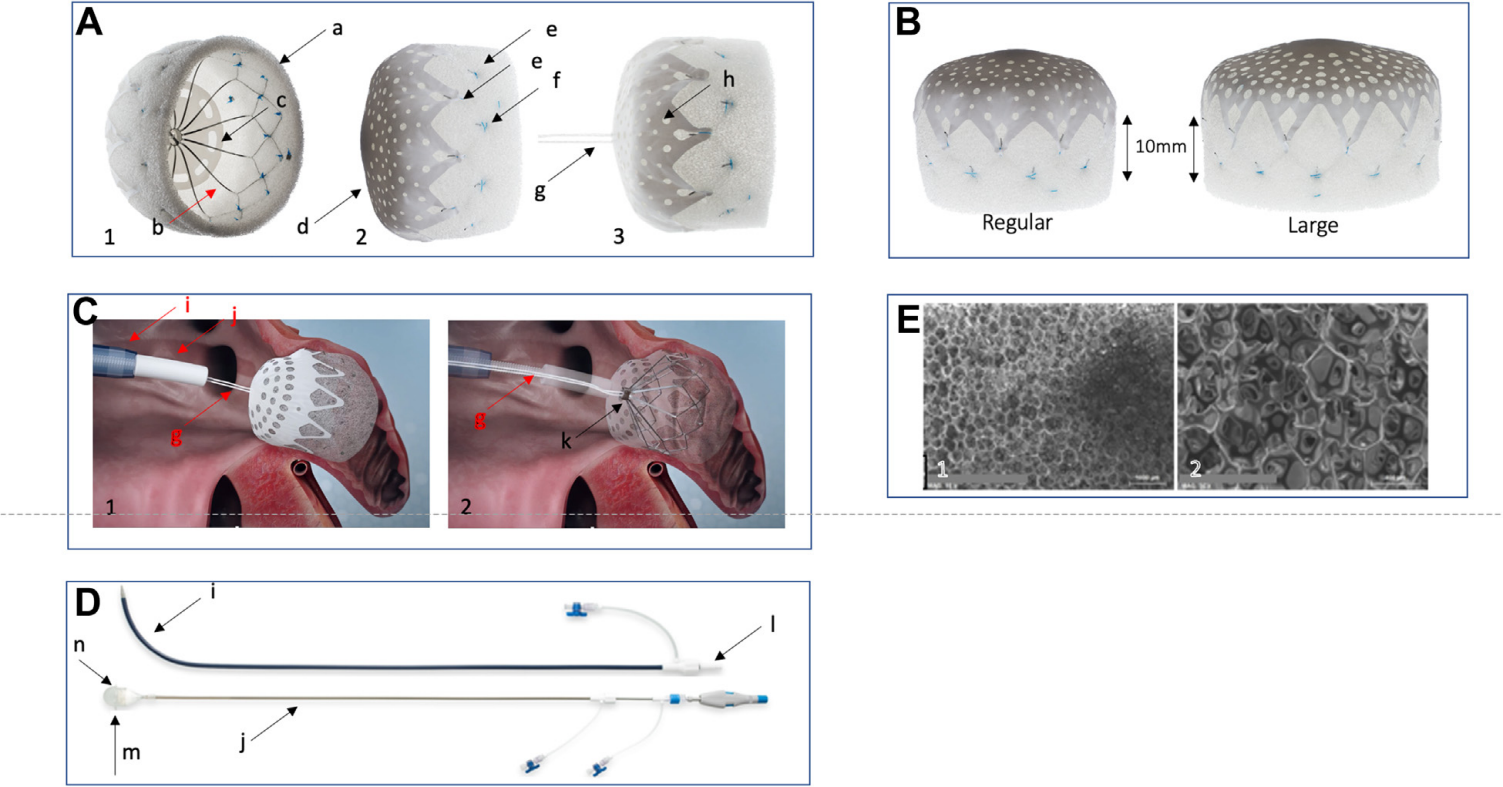
Conformal Left Atrial Appendage System (CLAAS) device. (**A**) Pictures of the CLAAS device in 3 projections, demonstrating key design elements including a foam cup (a) and an endoskeleton (b), which provides the outward structure and a platform for 2 rows of anchors (e). The CLAAS device has an external expanded polytetrafluoroethylene (ePTFE) cover (d) that has an array of fenestrations (f) and an internal ePTFE disc (c) positioned between the deep surface of the foam and the endoskeleton. The ePTFE cover is affixed to the endoskeleton by a series of sutures (f). Panel (**A-3**) is a side-view picture of the CLAAS device with the tether (g) in place. (**B**) Pictures of the CLAAS devices (regular and large) in a side view. Noted is the minimum depth required to anchor the device, which is 10 mm for both sizes. (**C**) Schematic of the CLAAS device in place following deployment before final tether release. Panel (**C-1**) shows the CLAAS device deployed within the left atrial appendage attached to the delivery system via the tether (g), which passes through the delivery catheter (j), which is positioned in the left atrium via the access sheath (i). Panel (**C-2**) shows schematic rendering with the access sheath, delivery system, ePTFE cover, and foam (transparent). Image demonstrates the tether (g) coursing through the delivery system in the implant and around the central post (not shown) within the endoskeleton nipple (k). (**D**) Pictures of the CLAAS access sheath (i) and delivery catheter (j). The access sheath is seen with dilator (l) in place ready for deployment across the intra-atrial septum. The lower picture shows the CLAAS delivery catheter (j), with the implant (n) within the loading cone (m). This assembly allows for the loading of the CLAAS device into the delivery catheter for deployment. (**E**) Scanning electron microscopy of the cross-linked, polyurethane polycarbonate-urea foam matrix cover (magnification: E-1, ×15 and E-2, ×50). Images show the highly porous open-cell structure of the foam matrix.

The CLAAS system comprises 2 components: the access sheath/dilator and the delivery catheter with implant, as shown in the Central Illustration. The access sheath is placed in the LAA following a transseptal puncture using standard techniques. The implant is loaded into the delivery catheter, flushed to remove air, and transferred to the LAA through the previously placed access sheath. The CLAAS system is available in 2 sizes—regular and large.

### Choice of animal model

The canine model was chosen because of its anatomic similarity to the human LAA and its historical use to assess other LAAO devices.^19^, ^20^, ^21^, ^22^, ^23^ Some have observed that the canine LAA has a constricted orifice and an angle of approach similar to those seen in humans.^19^

### Implantation procedure

All studies were performed at the American Preclinical Services. Study procedures were approved by the American Preclinical Services’ Institutional Animal Care and Use Committee. Eight mongrel dogs weighing between 25 and 45 kg were used in this study. Baseline assessments before the implant procedure included body weight and a baseline physical and neurological examination performed by a veterinarian within 3 days before the implant procedure. Animals were fasted for at least 12 hours before procedure induction. On day 0, animals received aspirin and nifedipine at standard doses before induction. General anesthesia was induced and maintained with isoflurane and propofol. Each animal received initial heparin doses of 50 IU/kg, with subsequent doses given as needed to maintain an activated clotting time between 250 and 400 seconds. Access to the LA was achieved via a right femoral vein puncture and a transseptal puncture. The CLAAS device was deployed into the LAA under echocardiographic and fluoroscopic guidance. Device position and seal were assessed by intracardiac echocardiography (ICE) and angiography. The access sheath was removed, after which the incision was closed using standard surgical techniques. Animals received incision scoring, physical examinations, neurological examinations, and body weight assessments.

### Postprocedure antiplatelet therapy

The postprocedure antiplatelet regime was chosen to reflect what is planned in the human clinical trials. Following device implantation, animals received aspirin (81 mg/d) and clopidogrel (75 mg/d) from day 1 until sacrifice.

### Follow-up methods

The follow-up evaluation consisted of sedated transthoracic echocardiography (TTE) performed on day 2 ± 1 and day 45 ± 5 after procedure. Before these procedures, the study animals were sedated with acepromazine. Follow-up TTE procedures assessed device stability, device location, evidence of thrombi or leaks, and the presence of pericardial effusion.

### Termination procedure methods

Animals were maintained for 90 (n = 4) or 150 (n = 4) days. Before termination, ICE and fluoroscopic examinations were performed to assess device stability, integrity, thrombi, leaks, pericardial effusion, and transseptal puncture site healing.

### Gross necropsy

Complete necropsies were performed, which included a gross examination of the external surface of the body, all orifices, and the thoracic and abdominal cavities and their contents for evidence of distal thromboembolism attributable to the test article. The heart was explanted and submitted for high-resolution radiographs (Faxitron [Hologic] images), allowing for the in situ examination of the implant before dissection, which included the evaluation of the implant surface and examination of its edges for leaks using a probe. The LAA with the implant in place underwent histologic sectioning and staining with hematoxylin and eosin. The other major organs were examined grossly, after which representative samples were collected for standard histologic examination.

## Results

### Implantation

A total of 8 animals underwent the CLAAS implantation procedure. All animals were in a normal sinus rhythm. The baseline ICE examination demonstrated structurally normal canine hearts without pericardial effusion or LAA thrombi (mean LAA ostium diameter: 15.2-21.5 mm; LAA depth: 16.0-23.0 mm). All LAAs were closed with the CLAAS implant (regular size), meeting the release criteria for seals with <5-mm leak, position in relation to the LAA ostium, and anchoring demonstrated by “Tug” testing, before the final tether release. In 3 animals, initial deployments did not meet the release criteria, requiring recapture and redeployment. After release, the ICE evaluation was repeated, which reconfirmed successful closure. In 1 animal, a leak of ∼3.0 mm was noted (Table 1). No pericardial effusions were noted. The access sheath was removed, and the access site was closed by standard surgical techniques. All animals were recovered without complications.

**Table 1:** Results of leak assessment.

Timepoint	Procedure	2 d	45 d	Presacrifice (90 d)	Gross examination (90 d)	Presacrifice (150 d)	Gross examination (150 d)
Method	ICE	TTE	TTE	ICE	Probe	ICE	Probe
Leaks^a^^,^^b^^,^^c^	1/8	0/8	0/8	1/4	0/4	0/4	0/4

ICE, intracardiac echocardiography; TTE, transthoracic echocardiography.

a All observed leaks were noted, not just the ones above a specific size.

b The animal observed to have a leak procedurally was not the same animal observed to have a leak presacrifice.

c The leak observed in 1 animal procedurally was a 3-mm leak on the posterior side of the implant; the leak observed in 1 animal presacrifice was an inferior posterior leak estimated at 2 to 3 mm.

### Follow-up echocardiographic assessment (2- and 45-day TTE and preterminal ICE)

Follow-up TTEs performed at 2 and 45 days demonstrated all devices to be stable, with no thrombi, leaks, or effusions, including in the animal in which a leak had been previously detected. Preterminal ICE and fluoroscopic examinations performed confirmed a stable device position and integrity in all animals. There were no device-related thrombi or pericardial effusions. In 1 animal sacrificed at 90 days, a leak (2-3 mm) was detected by ICE (Table 1). Of note, this was not the animal in which a leak was observed on the postprocedure examination.

### Gross necropsy

**Figure 1:**
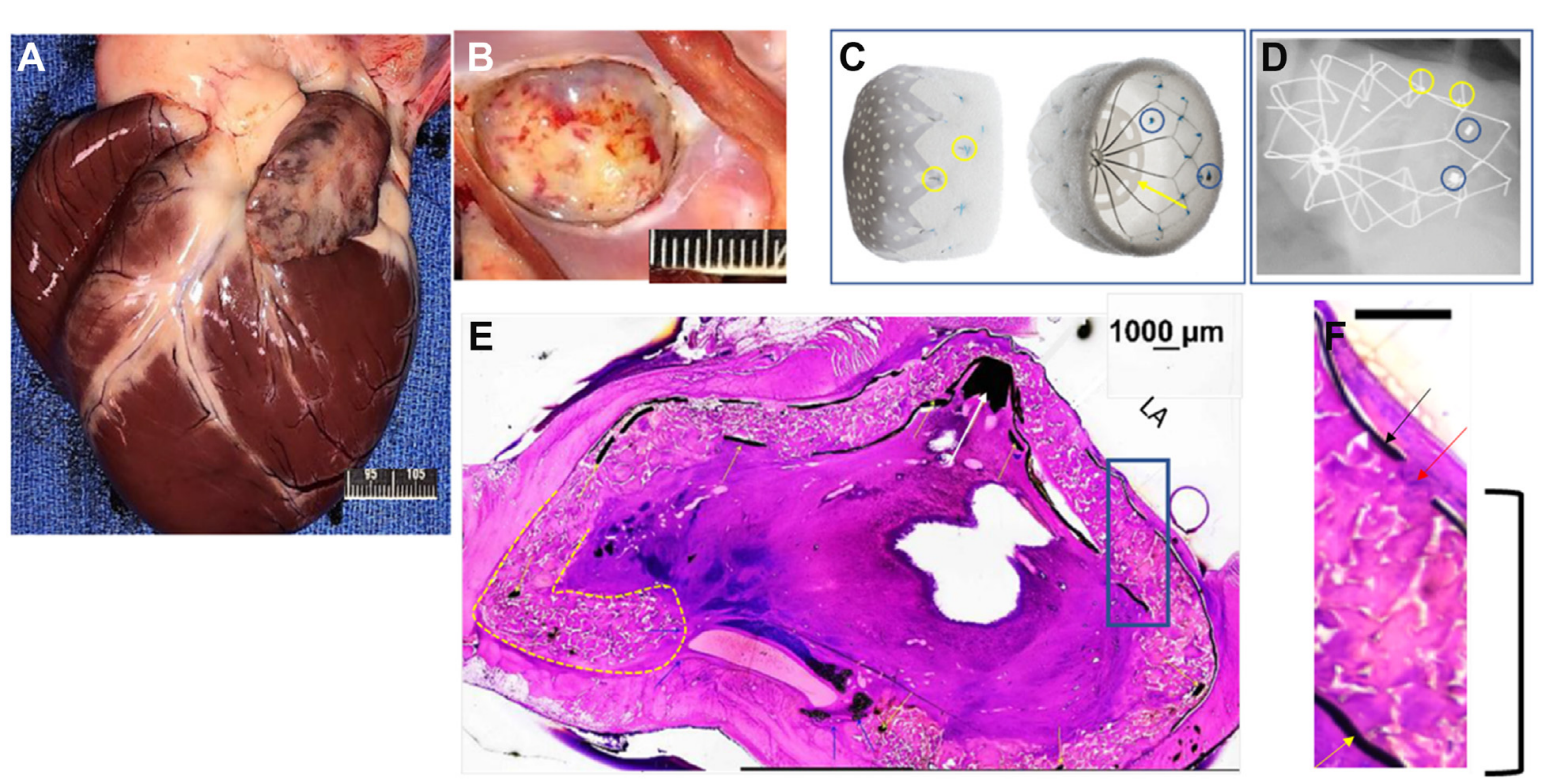
**90-Day animal: gross, histologic,and high-resolution x-ray examination.** Evaluation of the Conformal Left Atrial Appendage System (CLAAS) device implanted in the left atrial appendage (LAA) in a canine sacrificed 90 days after implantation. (**A**) Representative gross appearance of the anterior surface of the heart. The epicardial surface appears normal with minimal signs of inflammation. (**B**) Representative gross appearance of the LAA ostium with the CLAAS device in place. The device is covered with a yellow white material with red discoloration, consistent with normal healing. (**C**) Pictures of the CLAAS device in 2 projections, highlighting fluoroscopic markers (blue circles), anchors (yellow circles), and the internal expanded polytetrafluoroethylene (ePTFE) disk (yellow arrow). (**D**) Representative high-resolution x-ray images of the CLAAS devices in situ in an oblique projection, demonstrating an endoskeleton that is deformed to accommodate to the LAA anatomy. The endoskeleton is fully intact without fractures, with anchors deployed (yellow circles). Radiopaque markers are visible (blue circles). (**E**) The white arrows are pointing to the center of the implant which for most LAAC devices, needs to be situated co-axial within the ostium of the LAA, which can make delivery challenging. With the CLAAS device, it can be implanted off-axis and still seal the LAA, as seen here where the nipple (white arrow) is located adjacent to the edge of the ostium as opposed to in the center. The yellow lines surround the distal foam bumper of the implant. This feature it to protect the distal portion of the LAA from damage/performation by the device. That can be seen here where the distal portion of the foam folds over, protecting the LAA wall from direct contact with the distal end of the metallic scaffold. The center of the device is filled with an organized thrombus, with infiltrating macrophages and mononuclear cells. Scale bar: 1000 μm. (**F**) Enlarged portion of the hematoxylin and eosin–stained section. Implant delineated by the black box seen in panel E. The left atrium (LA) surface is covered by a thin layer of fibrotic material with rare monocytic cells infiltrated. The ePTFE cover is seen as a black linear element (black arrow) with fenestrations observed at regular intervals (red arrow). Deep into the ePTFE cover is the foam layer, which is seen as an amorphic substrate (black bracket). Deep into the foam is the internal ePTFE disk (yellow arrow). Scale bar: 1000 μm.

All animals survived to the prespecified interval of 90 (n = 4) and 150 (n = 4) days without signs of systemic illness, at which time they were sacrificed. The extracardiac examination demonstrated healthy animals without signs of systemic toxicity or infections. There were no focal signs on gross or histologic examination of embolism. Gross examination of the heart revealed the LAA epicardial surface and adjacent structures to have minimal inflammation, for example, thickening with few adhesions in the region of the device (Figure 1). In 4 animals (2-90 day and 2-150 day), a few anchors (∼2 anchors) were visible and palpable from the epicardial surface. There were no lacerations on the endocardium or epicardium.

No thrombotic material was observed within the LA or within the right and left ventricles.

**Figure 2:**
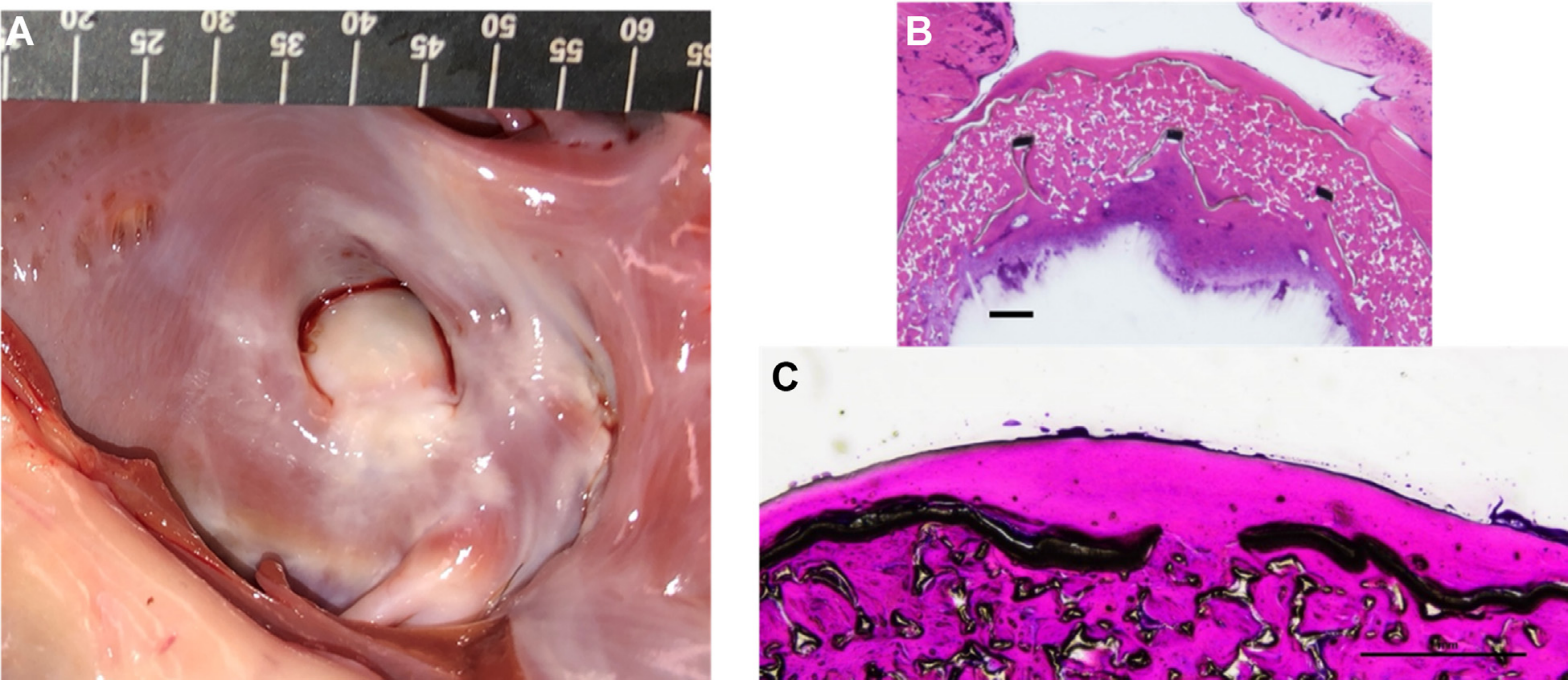
**150-Day animal: gross, histologic, and high-resolution x-ray examination.** (**A**) Representative gross appearance of the left atrial appendage (LAA) ostium with Conformal Left Atrial Appendage System (CLAAS) device in place, demonstrating complete coverage of the implant with a smooth fibrotic material. (**B**) Representative longitudinal/oblique section of the CLAAS device in situ, with the LAA stained with hematoxylin and eosin. A complete seal of the LAA can be seen, as well as the neointimal coverage. The outer expanded polytetrafluoroethylene (ePTFE) cover, the foam layer, as well as the inner ePTFE disc and segments of the nitinol endoskeleton, can be visualized. The foam material is primarily acellular, with occasional mononuclear and multinucleated giant cells noted. Scale bar: 1000 μm. (**C**) Magnified portion of the hematoxylin and eosin–stained section. The left atrium (LA) surface is covered by a thin layer of fibrotic material (red arrow). The ePTFE cover is seen as a black linear element (thin black arrow), with one of the fenestrations observed. Deep into the ePTFE is the foam layer, which is seen as an amorphic substrate. Scale bar: 1000 μm.

The LA surfaces of the CLAAS implants were diffusely covered by a layer of fibrosis (Figures 1 and 2). There were no visible leaks noted around the edge of the CLAAS devices at the appendage ostium. The left circumflex and left anterior descending arteries exhibited no gross abnormalities. The transseptal access sites were mildly fibrotic, which is considered normal following a transseptal puncture. High-resolution radiography performed on the CLAAS implants in situ showed that the device endoskeletons of all devices were deformed to accommodate the LAA anatomy, with all struts intact and without fractures.

### Histopathology

The histopathologic evaluation of extracardiac tissue from animals sacrificed at 90 and 150 days after implantation was without signs of systemic embolism, toxicity, or infection and showed fibrotic integration of an intact device without signs of endoskeletal fracture, foam breakdown, or significant inflammation (Figures 1 and 2). The devices at the level of the appendage ostium were covered by fibrosis lined with flattened endothelial-like cells with few foci of the exposed device fabric or fibrin thrombus material. In all these cases, the device completely sealed the appendage and was frequently positioned off axis compared with the long axis of the appendage. The distal leading edge of the device composed of foam was often folded on itself. The foam was primarily acellular, with occasional monocytic cells and multinucleated giant cells observed. The center of the device and the distal portion of the LAA were filled with organized thrombi with macrophages and mononuclear cells, multifocal aggregates/sheets of lymphocytes, and few polymorphonuclear cells. In addition, in the 150-day implants, multifocal osseous/chondroid metaplasia located centrally within the organizing thrombus was noted.

## Discussion

This study demonstrates that the CLAAS implant can successfully close the LAA in a healthy canine model. Sequential evaluation of the device using ultrasound, fluoroscopy, and necropsy shows the device to have maintained structural integrity, providing complete LAA closure with normal local healing without signs of systemic embolization, infection, or toxicity.

The use of a foam matrix to occlude vascular structures such as the LAA has been hindered by concerns regarding degradation following implantation and the lack of structural integrity precluding recapture without tearing. The CLAAS device was designed to address these concerns. The cross-linked polyurethane polycarbonate foam used in this device was selected because of its structure, which is resilient to the hydrolytic and enzymatic breakdown seen with other foams. In addition, the formulation is resistant to tearing. The ePTFE cover further increases the tear strength, allowing for the device to be recaptured into the access sheath without damage. Although others have examined the healing response to nitinol-frame LAAO devices that incorporate either polyethylene terephthalate or nonwoven polycarbonate urethane, this is the first long-term (>60 days) report of a new LAAO device that utilizes a novel reticulated, cross-linked, polyurethane polycarbonate-urea foam matrix.^14^^,^^19^, ^20^, ^21^, ^22^, ^23^, ^24^ The foam matrix enhances sealing, tissue adhesion to the LAA, and promotes fibrous neointima formation over the ePTFE cover and through its fenestrations. The folding of the foam at the distal edge validates the design intent to provide an atraumatic surface to minimize the risk of LAA damage or perforation and to facilitate complete closure and anchoring. All implants were noted to be visible and stable on ICE, TTE, and fluoroscopy. Although 2 animals were noted to have small (<5-mm) leaks on ultrasound evaluation at the implant procedure (n = 1) and at 90 days after implant and before sacrifice at the terminal procedure (n = 1), both were confirmed to be completely sealed on gross examination. These inconsistent findings highlight the inaccuracy of ultrasound evaluation in this model and the importance of gross examination.

## Study limitations

The study limitations include the small sample size, short duration of follow-up, and use of a healthy canine model. This model was selected due to similarities between the canine and human LAA anatomy. Furthermore, the canine model has been used to evaluate the majority of LAA closure devices that have been used clinically.^19^, ^20^, ^21^, ^22^, ^23^ Caution is required when extrapolating these results to clinical use.

## Conclusions

The gross and histologic evaluation of the CLAAS device in the chronic canine animal model at 90 and 150 days demonstrates definitive LAA occlusion and an appropriate healing response and provides the justification for moving into expanded clinical trials.^16^^,^^17^
